# The Effect of One Night of Sleep on Mnemonic Discrimination of Emotional Information

**DOI:** 10.3390/brainsci13030434

**Published:** 2023-03-02

**Authors:** Nicola Cellini

**Affiliations:** Department of General Psychology, University of Padova, Via Venezia 8, 35131 Padova, Italy; nicola.cellini@unipd.it

**Keywords:** emotional memory, lure discrimination, mnemonic discrimination, recognition memory, sleep

## Abstract

Mnemonic discrimination is the process of separating similar but distinct experiences and memories in the brain. This process seems to be differently modulated by retention periods that included sleep or only wakefulness. The current study was designed to explore whether a night of sleep may modulate emotional mnemonic discrimination. Thirty-eight participants performed the encoding phase of an emotional mnemonic similarity task composed of 48 negative, 48 neutral, and 48 positive pictures at 9 am (Wake group) or 9 pm (Sleep group). The encoding was followed by an immediate test (T1) in which participants were exposed to 36 targets (old), 36 foils (new), and 36 lures (similar) pictures, and they had to decide whether the pictures were old, new, or similar. Twelve hours later, they performed a delayed test (T2), similar to T1 but with different stimuli. Overall, performances decreased from T1 to T2. There were no differences between groups for recognition memories, whereas the reduction in mnemonic discrimination was more pronounced in the Wake group. Moreover, negative pictures were better discriminated that the neutral and positive ones. In conclusion, the current study showed that a night of sleep can stabilize mnemonic discrimination, regardless of the valence of the encoded stimuli, suggesting that sleep may not preferentially process emotional information.

## 1. Introduction

Pattern separation refers to a computational process of the hippocampus that separates similar but distinct experiences and memories in the brain [1,2]. This process is essential to encode distinct but similar events, such as remembering where we parked our car in a large parking lot. Pattern separation can be behaviorally measured by using tasks such as the Mnemonic Similarity Task (MST) [3,4]. In this task, participants have to correctly discriminate between items already encoded (target), items not previously seen (foils), and “lure” items (i.e., items similar to the target but different in terms of colors, orientation, etc.), usually by identifying each item as “old”, “new”, and “similar”, respectively. To successfully discriminate targets from lure items, participants need to create a detailed representation of these items. This ability to discriminate between similar items is commonly referred to as mnemonic discrimination [5]. For this specific task, it is usually assessed using the Lure Discrimination Index (LDI).

Recently, two studies have shown that sleep may play a crucial role in mnemonic discrimination [6,7]. Both studies showed that after a 12-h interval of either wakefulness or sleep, participants who slept had better mnemonic discrimination compared to participants who stayed awake. These studies suggest that sleep may help to stabilize (i.e., protect from interference) memories. Another study showed that performances at the MST task were lower after a sleep-deprived night compared to a regular night of sleep, but they were restored after a recovery 90-min nap [8]. Two studies have tested the effect of a daytime nap on mnemonic discrimination [9,10]. In both studies, the authors found that about 60–90 min of sleep did not facilitate mnemonic discrimination or recognition performance compared to a similar period of wakefulness.

These studies suggest that nocturnal sleep, but not daytime nap, may play a key role in mnemonic discrimination, at least for neutral stimuli. However, mnemonic discrimination can also occur for emotional events [11,12,13,14,15]. Indeed, separating similar but distinct emotional experiences is an important aspect of emotional regulation, allowing individuals to differentiate between similar events that have different emotional significance [16].

In 2014, Leal et al. [12] developed an emotional mnemonic similarity task directly derived from the original MST. In that study, two distinct groups of participants performed an incidental encoding of the task followed by either an immediate or 24-h delayed surprise test. At the immediate test, the authors observed a greater target recognition (*d’*) for negative and neutral pictures compared to the neural ones. At the same time, they observed lower discrimination of similar items for emotional pictures (positive and negative) compared to neutral ones. At the delayed test, target recognition was more preserved for emotional items than for neutral ones. Moreover, although the discrimination of similar stimuli decreased for all types of pictures, the forgetting of similar emotional items was more pronounced. This study seems to suggest that the negative stimuli tend to be easily encoded and harder to forget after a 24-h retention period, although these memories are more generic (e.g., we remember the gist) rather than specific (e.g., we forget non-salient details). It should be noted that his study participants could only provide “old” and “new” responses to the pictures, but not a “similar” response as in the original MST. These results were later replicated by the same [11,17] and other research groups [15], with the latter study also showing that the reported effect of emotion in mnemonic discrimination depends on the instruction provided (e.g., allowing a “similar” response) and the formula used to compute the main parameters. Indeed, when using the standard formula to compute the LDI (see [4]), emotional information shows a higher discriminability than neutral ones. A more recent study by Szőllősi and colleagues [14] tested the acute effect of stress (i.e., a socially evaluated cold pressor test 15-min before the task) on emotional mnemonic discrimination, replicating the greater discriminability of emotional stimuli compared to neutral stimuli. Moreover, they showed a positive relationship between cortisol response and mnemonic discrimination. Another study, using a different type of emotional stimuli, tested the effect of acute psychosocial stress on emotional mnemonic discrimination 24 h after the encoding [18]. The authors reported a higher recognition memory for negative stimuli compared to neutral stimuli in both the stress and control groups. They also observed a higher mnemonic discrimination for negative stimuli, but only in the stressed condition.

All in all, these studies are showing that emotional, in particular negative, information is better discriminated than neutral ones, either just after the encoding or 24 h later. However, although these “24-h retention periods” likely included some sleep, no study reported any measure of sleep quality or quantity.

As mentioned earlier, previous studies have already shown a facilitatory effect of sleep on mnemonic discrimination of neutral information [6,7,9,10]. This is in line with the general consensus of a key role of sleep in general memory retention [19,20]. However, the effect of sleep in selectively promoting the consolidation of emotional information is still debated. While early studies suggested that emotional memories, in particular of unpleasant and highly arousing information, were preferentially consolidated by sleep [21,22,23], recent reviews and meta-analyses indicate no preferential effect of sleep on emotional information over neutral information [24,25,26]. However, investigations on the effect of sleep on emotional memory discrimination are lacking.

Based on this literature, in the current study, I aimed to explore whether a night of sleep may facilitate the long-term discrimination of emotional information. Based on the literature showing a generally beneficial role of sleep in memory consolidation [27], and two studies showing a protective role of nocturnal sleep on mnemonic discrimination ability [6,7] for neutral stimuli, I expected to observe a greater mnemonic discrimination and recognition memory 12 h after encoding in participants who slept compared to those who remained awake throughout the day. Moreover, based on both the literature on sleep and emotional processing, and previous studies using the emotional version of the MST, I expect better memory retention and higher mnemonic discrimination for negative stimuli compared to neutral and positive stimuli, regardless of the sleep or wake condition [24,25,26,28].

## 2. Materials and Methods

### 2.1. Participants

Thirty-eight participants (22 F) with ages ranging from 18 to 25 years old participated in the study. Before the experimental session, participants were assigned to a Sleep group (n = 19, 13 F, Mage = 24.74 ± 3.51 years) or a Wake group (n = 19, 9 F, Mage = 25.31 ± 3.94 years) based on the order of recruitment. Participants reported to have no history of psychiatric or neurological disorders and having normal or corrected-to-normal vision. The study was approved by the Ethics Committee of the Departments of Psychology, University of Padova. All participants provided written consent before participation in this study.

### 2.2. Self-Reported Questionnaires

A few days before the experimental session, participants completed remotely-using Google form-a set of questionnaires to obtain basic demographics (age, gender, occupation) and investigate the perceived sleep quality, circadian preferences, anxiety, and depression levels. In detail, participants completed the Pittsburg Sleep Quality Index (PSQI; [29,30]) to assess subjective sleep quality. For the PSQI, a total score higher than 5 indicates the presence of poor sleep quality. From the PSQI, the Sleep Efficiency (SE, %), Sleep Latency (SOL, min), and Total Sleep Time (TST, min) indices were also extracted. The Morningness–Eveningness Questionnaire reduced version (rMEQ; [31,32]) was used to assess circadian preferences, with higher scores indicating a tendency to morning preferences. The Hospital Anxiety and Depression Scale (HAND-A and HADS-D; [33]) was used to assess anxiety and depression levels, and for each scale, scores between 11–14 and scores higher than 15 were considered as moderate or severe symptomatology, respectively [34].

Moreover, before the encoding and the delayed testing session (see below), participants’ sleepiness and fatigue levels were assessed using the Samn–Perelli Scale (SAMN; [35]) and the Stanford Sleepiness Scale (SSS; [36]), respectively.

### 2.3. Stimuli and Task

To investigate emotional pattern separation, we used the Emotional Memory Similarity Task [12], a task derived from the standard Mnemonic Similarity Task (MST) [3,4,37], which used emotional pictures ranging from highly pleasant to highly unpleasant, and from low to very arousing. This task has been designed to measure the ability to discriminate between pictures seen before and new pictures that are similar to those seen before, but these pictures can be categorized as “positive”, “neutral”, and “negative”.

**Figure 1:**
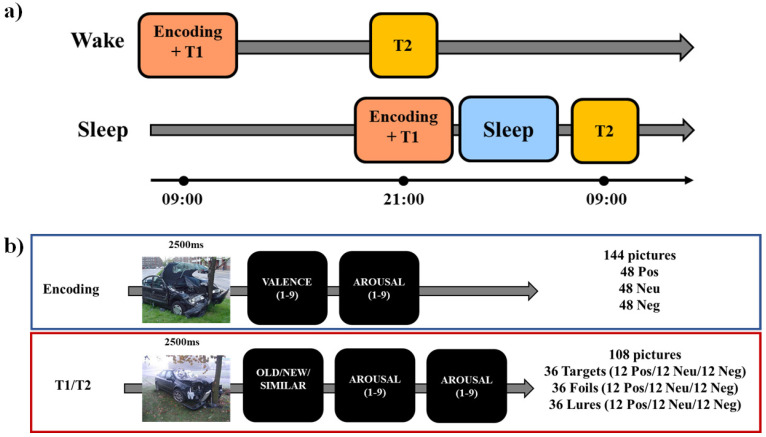
Schematic representation of (**a**) the experimental protocol and (**b**) the emotional mnemonic similarity task. Neg: Negative; Neu: Neutral; Pos: Positive. T1: immediate test. T2: delayed test (12-h later).

In the current study, each participant performed an encoding session and two testing sessions (immediate and delayed, Figure 1a). During the encoding phase, participants were exposed to 144 pictures (48 positive, 48 neutral, and 48 negative, Figure 1b). Each picture was presented for on the screen for 2500 ms, after which participants were asked to report on a nine-point scale their perceived ratings of valence (from very unpleasant to very pleasant) and arousal (from very calm to very arousing). To induce incidental learning, participants were not instructed to memorize the pictures but to look at them to provide the valence and arousal ratings. At the end of the encoding, participants were asked to wait 15 min before the next task, which was an immediate recognition task.

In each of the two test phases (immediate and delayed), participants were presented with 108 pictures: 36 targets (old), 36 foils (new), and 36 lures (similar to the targets). Targets were pictures presented at the encoding and foils were novel pictures, whereas lures were pictures “similar” to the ones presented at the encoding. For targets, foils, and lures, 12 pictures were negative, 12 neutral, and 12 positive. Lure items had two levels of similarity to targets (high and low similarity). In the testing phase, each picture was presented for 2500 ms, after which they were asked to respond using the mouse whether they had already seen it during the encoding (old) or not (new), or whether it was similar to one of the pictures presented at the encoding (similar). After that, they had to report their perceived ratings of valence and arousal. There was no time constraint for responding to each question (i.e., the test was self-paced). The pictures presented in the two testing phases were not the same (i.e., no stimulus was presented in both T1 and T2) but comparable in terms of balanced thematic content and arousal/valence levels. The use of two testing sessions was motivated by the need to have a baseline measure of the learning level for each participant before the 12-h retention period (either sleep or wake). This allows for measuring the individual change in memory retention after either a period of wakefulness or a period of sleep. Moreover, the use of different images in each testing session has been done to avoid repeated retrieval of the same information, which is known to affect long-term retention and subsequent testing [38,39,40,41]. The task has been built in PsychoPy3 [42], and testing was conducted using the online platform (Pavlovia, 2018).

For each participant, each testing session (T1 and T2), and each type of picture (negative, neutral, and positive), I computed the probability to respond “Old”, “New”, and “Similar” to the target, foils and lure. From these measures, I derived the Recognition Memory score (RM), calculated as *p* (old responses to target items)—*p* (old responses to foil items) to assess the ability to recognize target items. I also derived the Lure Discrimination Index (LDI), which measures the ability to create a different representation of similar presented items, which has been calculated following Stark and colleagues [3] method as *p* (similar responses to lure items)—*p* (similar responses to foil items). Lastly, to compare the current results to the ones by Leal and colleagues [11,12], I computed the bias-corrected LDI (bc-LDI) as *p* (new responses to lure items)—*p* (new responses to target items).

### 2.4. Procedure

For each participant, the whole experimental procedure was conducted remotely. Before the experimental session, all participants completed a set of questionnaires that included the PSQI, the MEQr, and the HADS. All testing sessions occurred between 8:00 and 10:00 AM and 8:00 and 10:00 PM (Figure 1a). In the “Wake” group, initial learning (encoding) took place at 09:00 ± 1:00, followed by an immediate memory test (T1), while the delayed test (T2) took place at 21:00 ± 1:00. In the “Sleep” group, the encoding and T1 were conducted at 21:00 ± 1:00, while T2 took place at 9:00 ± 1:00 on the following day after a night of sleep. Before the morning experimental task, participants completed a modified version of the PSQI [29] with a question related to the previous night, the SAMN [35], and the SSS [36], whereas the evening questionnaires before the task included only the SAMN and the SSS. All the tests were conducted online, with the experimenters calling the participants via ZOOM and remaining connected with them for the whole testing session.

### 2.5. Statistical Analysis

Demographic and trait variables were compared between groups using independent *t*-tests and the Chi-square test. For each comparison, we reported Cohen’s d as a measure of effect size.

The level of sleepiness, fatigue, and state anxiety at T1 and T2 has been analyzed using a mixed ANOVA 2 (Session: T1 and T2) × 2 (Group: Sleep and Wake).

The change in arousal and valence ratings between the two groups and across the sessions has been analyzed using a linear mixed model (LMM) with the type of image (negative, neutral, positive), session (Enc, T1, T2) and group (“Sleep” and “Wake”) as fixed effects, and participants as random effect. The change in memory performance between the two groups and across the sessions has been analyzed using an LMM with RM, LDI, or bc-LDI as the dependent variable, type of image (negative, neutral, positive), session (T1, T2) and group (“Sleep” and “Wake”) as fixed effects, and participants as random effect. For all the LMM, the Holm test was used for post-hoc analysis.

For these analyses, a *p* < 0.05 was used as the significant level.

The relationship between sleep characteristics and change in memory performance in RM and LDI (computed as the score at T2 minus the score at T1), as well as arousal and valence, was explored separately for the two groups using Pearson’s correlations. For the correlations, a *p* < 0.00083 was used as the significant level to account for multiple comparisons (Bonferroni correction).

All the analyses were conducted using JAMOVI 2.3 [43].

## 3. Results

### 3.1. Self-Reported Questionnaires

The two groups did not differ in demographic and trait variables and sleep habits (Table 1). No participants reported neither moderate nor severe depressive symptomatology, whereas three participants reported moderate symptomatology for anxiety.

### 3.2. Pre- and Post-Test Sleepiness and Fatigue Level

The analysis on fatigue showed a significant interaction Group × Session (F_1,36_ = 9.889, *p* = 0.003), but no significant post-hoc comparison emerged (all *p’s* > 0.073). At T2, fatigue was nominally higher in the “Wake” group than in the “Sleep” group (3.79 ± 1.40 vs. 2.84 ± 1.39, *p* = 0.088). No significant effect was observed for sleepiness (all *p’s* > 0.918).

### 3.3. Arousal and Valence ratings

**Figure 2:**
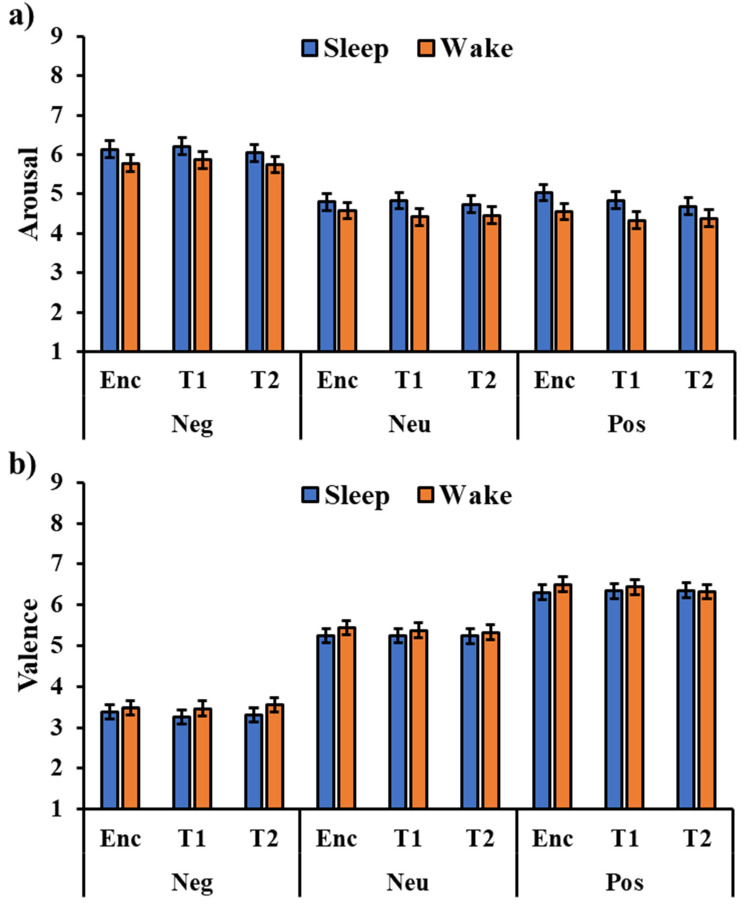
Arousal (**a**) and valence (**b**) ratings for the three types of images (Neg: Negative, Neu: Neutral, Pos: Positive) in the two groups in the encoding (Enc) and two testing sessions (T1, T2). The error bars represent the standard error of the means.

The analysis of the arousal ratings (Figure 2a) showed a significant effect of the type of image (F_2,288_ = 150.58, *p* < 0.001), with a higher arousal rating for negative pictures compared to the neutral (t_288_ = 15.03, *p_holm_* < 0.001) and positive ones (t_180_ = 15.03, *p_holm_* < 0.001). All the other effects or interactions were not significant (all *p’s* > 0.106), including the session’s main effect (F_2,288_ = 1.23, *p* = 0.293) and the Session × Type interaction (F_2,288_ = 0.51, *p* = 0.726), indicating that the valence rating was stable across testing sessions.

The analysis of valence ratings (Figure 2b) showed a significant type of image effect (F_2,288_ = 443.63, *p* < 0.001), with higher valence for the positive pictures compared to the neutral (t_288_ = 10.55, *p_holm_* < 0.001) and negative pictures (t_288_ = 29.40, *p_holm_* < 0.001), as well as a higher valence for the neutral pictures compared to the negative pictures (t_288_ = 18.85, *p_holm_* < 0.001). No other significant effect emerged (all *p’s* > 0.138), including the session’s main effect (F_2,288_ = 0.12, *p* = 0.891) and the Session × Type interaction (F_2,288_ = 0.06, *p* = 0.992), indicating that valence rating was stable across testing sessions.

### 3.4. Mnemonic Performance

**Figure 3:**
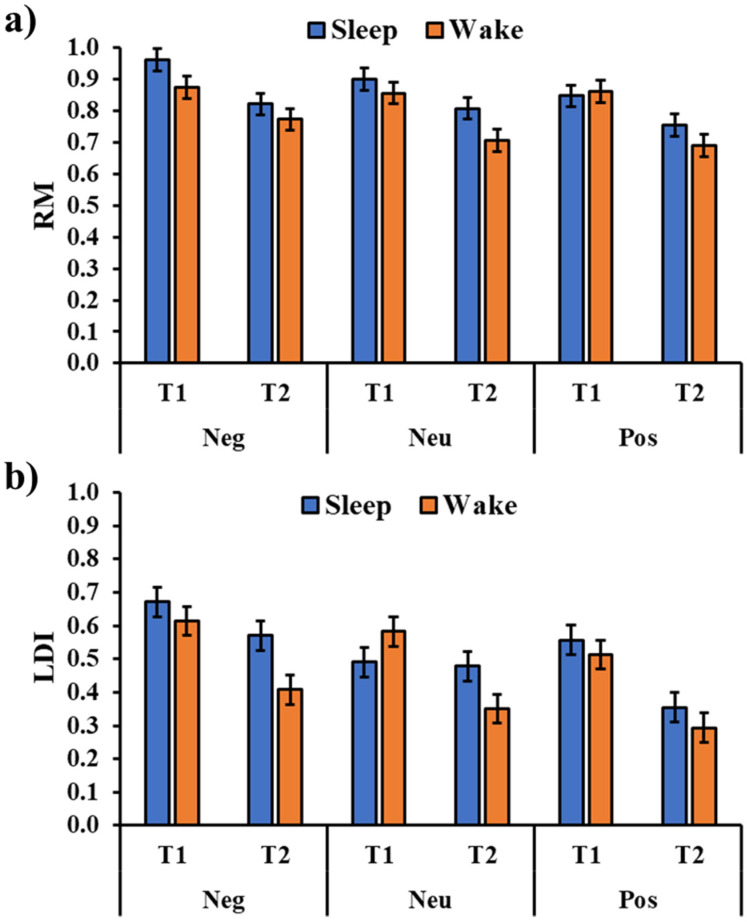
Recognition memory (RM, (**a**)) and Lure Discrimination Index (LDI, (**b**)) for the three types of images (Neg: Negative, Neu: Neutral, Pos: Positive) in the two groups and the two sessions (T1, T2). The error bars represent the standard error of the means.

The analysis of the RM (Figure 3a) showed a significant session main effect (F_1,180_ = 59.80, *p* < 0.001) with a significant reduction in the performance from T1 to T2. Moreover, the analysis showed a significant type of image main effect (F_2,180_ = 21.20, *p* = 0.002) with a greater RM Negative picture compared to a positive picture (t_180_ = 3.51, *p_holm_* = 0.002) and, although not reaching the statistical significance, a neutral picture (t_180_ = 2.00, *p_holm_* = 0.093). There was no significant effect for the group main effect (F_1,36_ = 2.51, *p* = 0.122), and the critical interactions Group × Type (F_2,180_ = 0.834, *p* = 0.436), Group × Session (F_1,180_ = 1.00, *p* = 0.950), and Group × Type × Session (F_2,180_ = 1.28, *p* = 0.280; Figure 3a).

**Figure 4:**
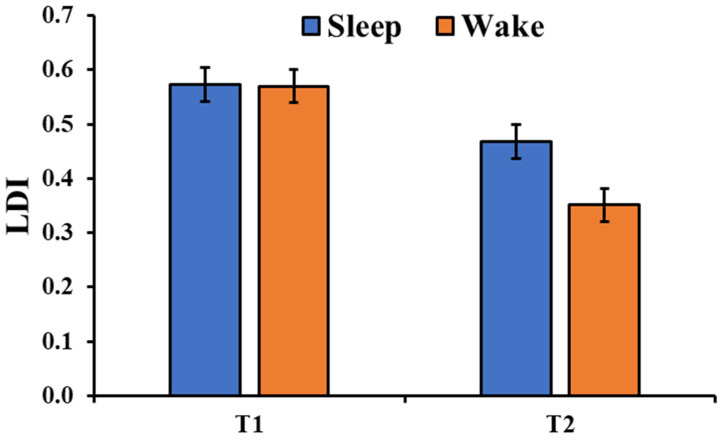
Lure discrimination index (LDI) in the two groups and the two sessions (T1, T2). The error bars represent the standard error of the means.

The analysis of the LDI (Figure 3b) showed a significant session main effect (F_1,180_ = 54.79, *p* < 0.001) with a significant performance decrement from T1 to T2. Moreover, there was a significant type of image main effect (F_2,180_ = 13.27, *p* < 0.001) with a higher mnemonic discrimination for the negative pictures compared to both the neutral (t_180_ = 3.35, *p_holm_* = 0.002) and positive pictures (t_180_ = 5.06, *p_holm_* < 0.001). Although the main effect of the group was not significant (F_1,36_ = 2.60, *p* = 0.116), there was a significant Group × Session interaction (F_1,180_ = 6.76, *p* = 0.010, Figure 4) with a greater discrimination memory after a period of sleep compared to a similar period of wakefulness (t_180_ = 2.71, *p_holm_* = 0.026). The critical interactions Group × Type (F_2,180_ = 1.499, *p* = 0.226) and Group × Type × Session (F_2,180_ = 1.774, *p* = 0.173) were not significant.

Lastly, the analysis of the bc-LDI showed only a significant session main effect (F_1,180_ = 20.37, *p* < 0.001) with a significant performance decrement from T1 to T2. The group (F_1,36_ = 1.82, *p* = 0.185) and type of image main effects (F_1,180_ = 1.15, *p* = 0.320), as well as the interactions Group × Type (F_2,180_ = 1.12, *p* = 0.330), Group × Session (F_1,180_ = 0.49, *p* = 0.483), Session × Type (F_2,180_ = 1.10, *p* = 0.336), and Group × Type × Session (F_2,180_ = 0.23, *p* = 0.799) were not significant.

### 3.5. Responses to High- and Low-Similarity Pictures

**Figure 5:**
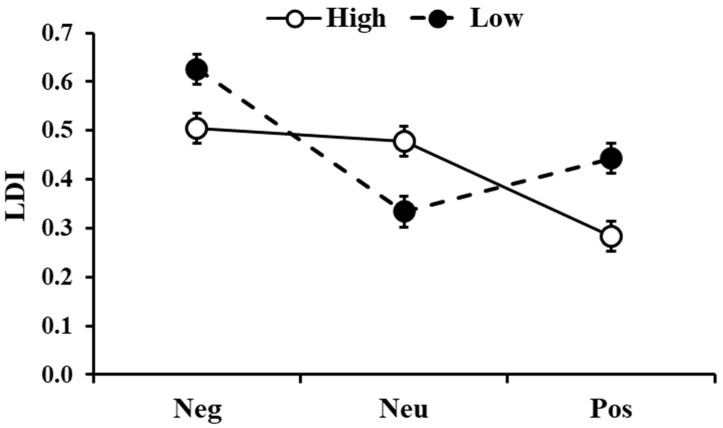
Lure discrimination index (LDI) as a function of the type of images (Neg: Negative, Neu: Neutral, Pos: Positive) and the similarity level (High and Low). The error bars represent the standard error of the means.

The analysis on the LDI, comparing pictures with low and high similarity, showed a significant effect of similarity (F_1,396_ = 4.057, *p* = 0.045) with low-similarity pictures being better discriminated than the high-similarity ones. There was also significant Similarity ×Type interactions (F_2,396_ = 17.83, *p* < 0.001, Figure 5) with higher LDI for negative and positive pictures in the low-similarity condition compared to the high-similarity condition (t_396_ = 3.09, *p_holm_* = 0.013 and t_396_ = 4.07, *p_holm_* < 0.001, respectively), whereas for neutral stimuli, LDI was higher for high-similarity compared to the low-similarity condition (t_396_ = 3.68, *p_holm_* = 0.002). In addition, for stimuli with low similarity, negative pictures showed a higher LDI than the neutral and positive ones (t_396_ = 7.47, *p_holm_* < 0.001 and t_396_ = 4.69, *p_holm_* < 0.001, respectively). For the stimuli with high similarity, LDI was higher for both negative and neutral pictures compared to the positive ones (t_396_ = 5.68, *p_holm_* < 0.001 and t_396_ = 4.97, *p_holm_* < 0.001, respectively).

### 3.6. Correlational Analysis

The correlational analyses did not show any significant association between sleep parameters and study variables in the two groups (see Supplementary Materials Tables S1 and S2).

## 4. Discussion

The current study was designed to explore whether nocturnal sleep could facilitate the long-term discrimination of emotional information. Based on the literature showing a generally beneficial role of sleep in memory consolidation [27] and two studies showing a protective role of nocturnal sleep on mnemonic discrimination ability [6,7] for neutral stimuli, I expected to observe a greater mnemonic discrimination and recognition memory 12 h after encoding in participants who slept compared to those who remained awake across the day.

As expected, the performance decreased between sessions in both conditions. For the recognition memory (RM), this decrease was not significantly different between the groups, and this result is in line with previous findings from Doxey and colleagues [6], and with the results of the two studies testing the effect of a daytime nap on memory discrimination [9,10]. The results also indicate that recognition was greater for negative stimuli compared to neutral and positive stimuli. This result is consistent with the literature suggesting that negative events are more likely to be encoded and retrieved than other types of events [44,45,46,47]. Moreover, this data is in line with a previous study using the same emotional task [12,15]. It is worth noting that there was no interaction between the group and session, indicating that sleep did not preferentially promote greater recognition of the negative stimuli. However, overall, negative pictures were better encoded and remembered. This is consistent with some recent reviews and meta-analyses indicating no preferential sleep-related effect of emotional information over neutral information [24,25,26].

Focusing on mnemonic discrimination, assessed here using the Lure Discrimination Index (LDI), the results indicate, as expected, a decrease performance across sessions. However, this decrease was reduced in the “Sleep” group compared to the “Wake” group. This result is in line with previous findings showing that mnemonic discrimination was greater after a night of sleep compared to a similar period of wakefulness [6,7], suggesting a role of sleep in protecting memories from interference. Here, data suggest that this sleep effect can occur regardless of the emotional valence of the stimuli. The analysis of LDI also indicates that, overall, the mnemonic discrimination was higher for negative stimuli compared to both the neutral and positive stimuli. This result partially replicates previous data from Szőllősi and colleagues [14,15], who showed a clear modulation of the arousal level in lure discrimination. Specifically, they reported a linear decrease in the LDI from negative to positive to neutral stimuli. Here, positive and neutral stimuli were discriminated similarly. This result may be related to the way participants rated their subjective arousal while watching the pictures. Indeed, in the current study, participants rated similarly their arousal levels for positive and neutral stimuli. In addition, the results of the bc-LDI are in line with Leal et al. [13] and Szőllősi and colleagues [15]. Indeed, although there was not a significant main effect of the valance when considering both tests (T1 and T2), the analysis of T1, which resembles the immediate test used in the mentioned studies, showed a trend for a valence main effect (F_2,72_ = 2.826, *p* = 0.066), with the rate of new responses for the lures that were higher for the neutral pictures compared to negative (*p* = 0.037) and positive pictures (*p* = 0.051).

The greater discrimination for the negative stimuli can be explained by taking into account the Negative Emotional Valence Enhances Recapitulation (NEVER) model proposed by Bowen and colleagues [28]. According to the NEVER model, negative stimuli enhance sensory-focused encoding, allowing the creation of a more detailed representation of the event. Moreover, the model proposes that negative materials are reactivated during offline periods (e.g., quiet wake or sleep), allowing for a stronger and preferential consolidation of this information.

Regarding emotional ratings, both perceived valence and arousal remained stable across the testing sessions. This result is partially in contrast to the “Sleep to Forget Sleep to Remember” model [48], which proposed that sleep promotes a decoupling between the memory content of an emotional event and the related affective tone. While the content is preserved over periods of sleep, the affective tone should start to decline after a sleep period. However, it is in line with the idea that sleep tends to preserve the emotional reactivity associated with an emotional event [49] and in line with previous empirical studies using subjective reports [50,51,52,53,54]. Interestingly, the current sample showed no difference in terms of arousal for neutral and positive stimuli. This was indeed surprising since the ratings for negative and positive stimuli were in line with previous studies using the current set of pictures (e.g., [12,15]), but neutral pictures were rated one point higher than in previous studies. I have no strong explanation for this result. I can only speculate that, in this study, participants assessed neutral and positive pictures as “non-negative”, therefore shifting their arousal rating away from the “negative” response side, landing of a mid-scale (i.e., the overall rating for positive and neutral was about 4.8 out of 9 for both of them). Another possibility is related to cultural differences. The set of images was validated in the US [12] and then in Hungary [15], but this is the first study using these pictures with an Italian sample. Future studies should aim to replicate this finding and provide normative ratings for these pictures in the Italian population.

The current results should be interpreted taking several limitations in mind. First, in the current study, sleep was assessed using self-reports. Second, the current study may have a low power to detect the main effect of interest. Indeed, the sample was of relatively low size, although in line with previous studies investigating the effect of sleep on the MST [6,7,10] and studies using the emotional version of the MST [11,12,13]. Moreover, the study used a between-subjects design, although it was designed to test participants’ performance both in immediate and delayed sessions. Therefore, future studies should replicate these findings using objective sleep assessment (i.e., polysomnography), increasing the sample size and trying to create a suitable task for a within-subjects design.

In conclusion, the current explorative study showed that a night of sleep can stabilize mnemonic discrimination, regardless of the valence of the encoded stimuli, suggesting that sleep may not preferentially process emotional information. Moreover, this study confirms previous investigations showing that negative stimuli are better encoded and discriminated compared to positive and neutral information.

## Figures and Tables

**Table 1:** Mean ± standard deviation of self-report questionnaires in the two groups.

	Sleep	Wake	t	p	Cohen’s d
Age (years)	24.74 ± 3.51	25.31 ± 3.94	−0.478	0.635	−0.155
Gender (F/M)	13/6	9/10	1.727 *	0.189	
MEQr	15.05 ± 4.74	14 ± 3.89	0.748	0.459	0.243
HADS-D	4.63 ± 2.93	5.16 ± 3.40	−0.511	0.613	−0.166
HADS-A	8.58 ± 3.32	7.63 ± 3.17	0.900	0.374	0.292
PSQI	6.47 ± 3.08	5.79 ± 2.55	0.746	0.461	0.242
TST (h)	7.03 ± 1.03	7.03 ± 0.98	0.001	0.999	0.001
SOL (min)	28.92 ± 20.84	20.53 ± 19.21	1.291	0.205	0.419
SE (%)	87.98 ± 10.11	92.54 ± 8.87	−1.477	0.148	−0.479

Notes. PSQI = Pittsburg Sleep Quality Index; MEQr = Morniness-Eveningness Questionnaire reduced version; HADS-A = anxiety scale of the Hospital Anxiety and Depression Scale; HADS-D = depression scale of the HADS; TST = total sleep time; SOL = sleep onset latency; SE= sleep efficiency. * = χ2 value.

## Data Availability

The data that support the findings of this study are available from the corresponding author upon reasonable request. The study was not preregistered.

## Supplementary Materials

The following supporting information can be downloaded at: https://www.mdpi.com/article/10.3390/brainsci13030434/s1, Table S1: Correlations between self-reported sleep parameters of the night between T1 and T2 and change in study variables in the Sleep group (n = 18); Table S2: Correlations between self-reported sleep parameters of the night before T1 and change in study variables in the Wake group (n = 18).
