# Supplementary material for: The Effect of One Night of Sleep on Mnemonic Discrimination of Emotional Information
Source: Brain Sci. 2023 Mar 2;13(3):434. doi: 10.3390/brainsci13030434 (PMC10046774; doi:10.3390/brainsci13030434)
Supplement: Supplementary file 1 [file brainsci-13-00434-s001.zip › brainsci-2203741-supplementary.pdf]

Supplemental Material

Table S1. Correlations between self-reported sleep parameters of the night between T1 and T2 and change in study variables in the Sleep group (n=18).

		ΔRM Neg	ΔRM Neu	ΔRM Pos	ΔLDI Neg	ΔLDI Neu	ΔLDI Pos	ΔAr Neg	ΔAr Neu	ΔAr Pos	ΔVal Neg	ΔVal Neu	ΔVal Pos
BT	r	-0.039	-0.110	-0.334	0.324	-0.174	0.060	-0.317	-0.117	-0.066	0.115	-0.019	0.184
	p	0.874	0.654	0.163	0.176	0.476	0.806	0.187	0.635	0.789	0.639	0.938	0.452
WT	r	-0.230	-0.194	-0.165	-0.119	0.106	-0.011	-0.201	0.068	0.239	0.486	0.201	-0.394
	p	0.345	0.427	0.500	0.628	0.667	0.966	0.410	0.782	0.323	0.035	0.409	0.095
TIB	r	-0.293	-0.363	-0.468	-0.064	-0.026	-0.231	-0.540	-0.053	0.083	0.551	0.279	-0.121
	p	0.224	0.127	0.043	0.794	0.916	0.342	0.017	0.829	0.735	0.015	0.248	0.622
TST	r	-0.170	-0.072	-0.535	-0.071	-0.095	-0.417	-0.451	0.234	0.090	0.429	0.124	-0.003
	p	0.485	0.769	0.018	0.774	0.700	0.076	0.053	0.335	0.715	0.067	0.612	0.992
SE	r	0.192	0.461	-0.142	0.008	-0.125	-0.322	0.087	0.440	0.031	-0.150	-0.181	0.177
	P	0.430	0.047	0.562	0.973	0.609	0.178	0.724	0.059	0.898	0.541	0.459	0.469

Notes. BT: Bedtime; WT: Waketime; TIB: Time in Bed; TST: Total Sleep Time; SE: Sleep Efficiency; RM: Recognition Memory; LDI: Lure Discrimination Index; Ar: Arousal; Val: Valence; Neg: Negative; Neu: Neutral; Pos: Positive. R: Pearson's r. p: uncorrected p-value. Using Bonferroni correction, the significant level is set to $p<0.00083$.

Table S2. Correlations between self-reported sleep parameters of the night before T1 and change in study variables in the Wake group (n=18).

		ΔRM Neg	ΔRM Neu	ΔRM Pos	ΔLDI Neg	ΔLDI Neu	ΔLDI Pos	ΔAr Neg	ΔAr Neu	ΔAr Pos	ΔVal Neg	ΔVal Neu	ΔVal Pos
BT	p	0.196	0.372	-0.193	0.036	-0.184	0.088	0.249	0.371	-0.022	0.033	-0.269	-0.174
	r	0.422	0.117	0.428	0.884	0.451	0.721	0.303	0.118	0.928	0.894	0.265	0.477
WT	p	0.613	-0.220	0.248	-0.361	-0.363	0.229	-0.346	-0.134	-0.169	0.343	0.126	-0.327
	r	0.005	0.366	0.307	0.129	0.127	0.345	0.147	0.585	0.490	0.151	0.606	0.171
TIB	p	0.318	0.087	-0.225	-0.122	-0.373	-0.016	0.208	0.091	-0.409	0.129	-0.179	-0.208
	r	0.185	0.724	0.354	0.619	0.115	0.947	0.393	0.712	0.082	0.599	0.465	0.393
TST	p	0.429	-0.055	-0.106	-0.036	-0.297	0.123	0.144	0.122	-0.271	0.297	-0.008	-0.193
	r	0.067	0.823	0.667	0.885	0.216	0.617	0.556	0.620	0.261	0.216	0.974	0.429
SE	P	0.178	-0.280	0.348	0.189	0.188	0.372	-0.266	0.043	0.412	0.309	0.379	0.109
	r	0.465	0.245	0.145	0.439	0.440	0.117	0.271	0.861	0.080	0.198	0.109	0.657

Notes. BT: Bedtime; WT: Waketime; TIB: Time in Bed; TST: Total Sleep Time; SE: Sleep Efficiency; RM: Recognition Memory; LDI: Lure Discrimination Index; Ar: Arousal; Val: Valence; Neg: Negative; Neu: Neutral; Pos: Positive. R: Pearson's r. p: uncorrected p-value. Using Bonferroni correction, the significant level is set to p<0.00083.
